# Prevalence and malignancy risk of focal colorectal incidental uptake detected by ^18^F-FDG-PET or PET/CT: a meta-analysis

**DOI:** 10.2478/raon-2013-0035

**Published:** 2014-04-25

**Authors:** Giorgio Treglia, Silvia Taralli, Marco Salsano, Barbara Muoio, Ramin Sadeghi, Luca Giovanella

**Affiliations:** 1 Department of Nuclear Medicine and PET/CT Center, Oncology Institute of Southern Switzerland, Bellinzona, Switzerland; 2 Institute of Nuclear Medicine, Catholic University of the Sacred Heart, Rome, Italy; 3 Institute of Radiology, Catholic University of the Sacred Heart, Rome, Italy; 4 School of Medicine, Catholic University of the Sacred Heart, Rome, Italy; 5 Nuclear Medicine Research Center, Mashhad University of Medical Sciences, Mashhad

**Keywords:** PET/CT, fluorodeoxyglucose, colonic uptake, incidentaloma, focal uptake, colorectal cancer

## Abstract

**Background:**

The aim of the study was to meta-analyze published data about prevalence and malignancy risk of focal colorectal incidentalomas (FCIs) detected by Fluorine-18-Fluorodeoxyglucose positron emission tomography or positron emission tomography/computed tomography (^18^F-FDG-PET or PET/CT).

**Methods:**

A comprehensive computer literature search of studies published through July 31^st^ 2012 regarding FCIs detected by ^18^F-FDG-PET or PET/CT was performed. Pooled prevalence of patients with FCIs and risk of malignant or premalignant FCIs after colonoscopy or histopathology verification were calculated. Furthermore, separate calculations for geographic areas were performed. Finally, average standardized uptake values (SUV) in malignant, premalignant and benign FCIs were reported.

**Results:**

Thirty-two studies comprising 89,061 patients evaluated by ^18^F-FDG-PET or PET/CT were included. The pooled prevalence of FCIs detected by ^18^F-FDG-PET or PET/CT was 3.6% (95% confidence interval [95% CI]: 2.6–4.7%). Overall, 1,044 FCIs detected by ^18^F-FDG-PET or PET/CT underwent colonoscopy or histopathology evaluation. Pooled risk of malignant or premalignant lesions was 68% (95% CI: 60–75%). Risk of malignant and premalignant FCIs in Asia-Oceania was lower compared to that of Europe and America. A significant overlap in average SUV was found between malignant, premalignant and benign FCIs.

**Conclusions:**

FCIs are observed in a not negligible number of patients who undergo ^18^F-FDG-PET or PET/CT studies with a high risk of malignant or premalignant lesions. SUV is not reliable as a tool to differentiate between malignant, premalignant and benign FCIs. Further investigation is warranted whenever FCIs are detected by ^18^F-FDG-PET or PET/CT.

## Introduction

Colorectal incidentalomas (CIs) are defined as unexpected colorectal findings that are discovered on an imaging study unrelated to the large bowel. CIs represent a challenge for the clinicians: some of these findings are benign but the risk of malignancy in CIs might be significant.^1^

As Fluorine-18-Fluorodeoxyglucose positron emission tomography or positron emission tomography/computed tomography (^18^F-FDG-PET or PET/CT) are increasingly used, especially for oncologic patients, incidental uptake detected by these functional imaging methods are also increasing. ^18^F-FDG-PET or PET/CT may sometimes reveal an unexpected area of increased radiopharmaceutical uptake within the large bowel in patients referred for other diseases and this finding is defined as CI.^1^,^2^

Both focal, segmental and diffuse unexpected ^18^F-FDG uptake in the large bowel were reported. Segmental and diffuse increased uptake of ^18^F-FDG in the large bowel are considered at low risk of malignancy, being more likely associated with inflammation, physiological uptake or radiopharmaceutical excretion. Conversely, unexpected focal ^18^F-FDG uptake in the large bowel is of greater concern since it may represent both benign, pre-malignant (*i.e.* colonic adenomas) or malignant lesions (*i.e.* primary colorectal cancer or metastatic lesions).^1^,^2^

Several articles have reported data about the prevalence and the malignancy risk of focal colorectal incidental uptake (FCIs) detected by ^18^F-FDG-PET or PET/CT with discordant results. A systematic review about this topic and a meta-analysis providing pooled estimates of prevalence and malignancy risk of FCIs detected by ^18^F-FDGPET or PET/CT are still lacking. Therefore, the objective of our article is to meta-analyze published data about prevalence and malignancy risk of FCIs detected by ^18^F-FDG-PET or PET/CT, in order to derive more robust estimates in this regard.

## Methods

### Search strategy

A comprehensive computer literature search of the PubMed/MEDLINE and Scopus databases was conducted to find relevant published articles on the prevalence and malignancy risk of FCIs detected by ^18^F-FDG-PET or PET/CT. We used a search algorithm that was based on a combination of the terms: “incidental” AND “PET” OR “positron emission tomography” OR “fluorodeoxyglucose” OR “FDG”. No beginning date limit was used; the search was updated until July 31^st^, 2012. Only articles in English language were selected. To expand our search, references of the retrieved articles were also screened for additional studies.

### Study selection

Original articles investigating both the prevalence and the malignancy risk of FCIs detected by ^18^F-FDG-PET or PET/CT were eligible for inclusion. The exclusion criteria were: a) articles not providing information about prevalence or malignancy risk of FCIs detected by ^18^F-FDG-PET or PET/CT; b) articles not in English language; c) overlap in patient data (in this case the most complete article was included). Three researchers independently reviewed the titles and abstracts of the retrieved articles, applying the inclusion and exclusion criteria mentioned above. Articles were rejected if they were clearly ineligible. The same three researchers then independently reviewed the full-text version of the remaining articles to determine their eligibility for inclusion.

### Data extraction

For each included study, information was collected concerning basic study data (authors, year of publication, country), instrumentation used (PET or PET/CT), number of patients evaluated with PET or PET/CT, number of FCIs detected by PET or PET/CT, number of FCIs verified by colonoscopy or histology, final diagnosis of FCIs, average standardized uptake values (SUV) in malignant, premalignant and benign FCIs.

### Statistical analysis

The prevalence of patients with FCIs who underwent PET or PET/CT was obtained from individual studies using this formula: prevalence of FCIs = number of patients with FCIs / number of patients evaluated with PET or PET/CT ×100.

The risk of malignant or premalignant FCIs detected by PET or PET/CT was obtained from individual studies using this formula: risk of malignant or premalignant FCIs = number of malignant or premalignant lesions found between FCIs / number of FCIs revealed by PET or PET/CT and verified by colonoscopy or histology ×100.

Patients with a history of colorectal cancer were excluded from the analysis.

A random-effects model was used for statistical pooling of the data; pooled data were presented with 95% confidence intervals (95% CI) and displayed using forest plots. A I-square statistic was also performed to test for heterogeneity between studies. A sub-analysis of the risk of malignant and premalignant FCIs taking into account different geographic areas was carried out. Statistical analyses were performed using StatsDirect statistical software version 2.7.9 (StatsDirect Limited, UK).

## Results

The comprehensive computer literature search from PubMed/MEDLINE and Scopus databases revealed 519 articles. Reviewing titles and abstracts, 492 articles were excluded because they did not report any data on prevalence neither on malignancy risk of FCIs detected by ^18^F-FDG-PET or PET/CT. One article was excluded because not in English language.^3^

**FIGURE 1.**
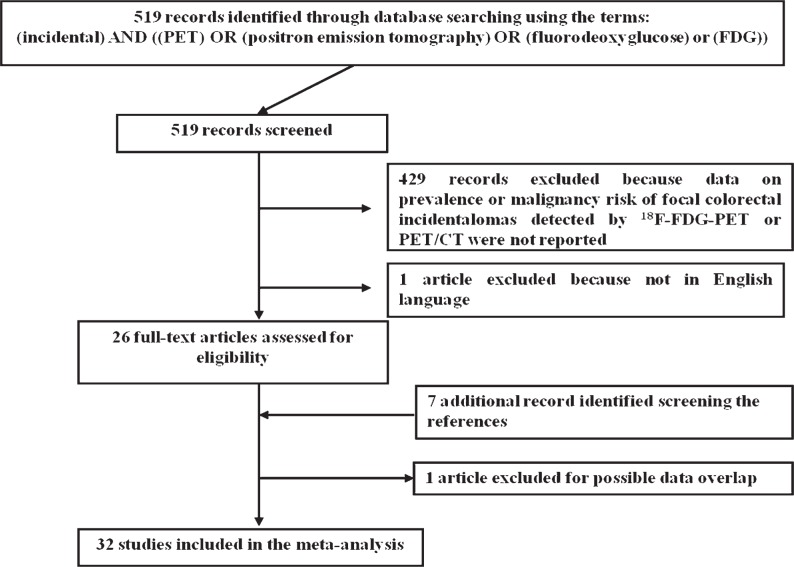
Flow chart of the search for eligible studies on the prevalence or malignancy risk of focal colorectal incidental uptake detected by ^18^F-FDG-PET or PET/CT.

Twenty-six articles were selected and retrieved in full-text version; seven additional studies were found screening the references of these articles. Out of these 33 articles potentially eligible for inclusion, after reviewing the full-text article, one article was excluded due to possible data overlap.^4^ Finally, 32 studies including 89,061 patients met all inclusion and exclusion criteria, and they were included in our meta-analysis (Figure 1) 2,5–35; 18 studies had data to calculate the pooled prevalence of FCIs and 31 studies had data to calculate the pooled risk of malignant of premalignant FCIs. The characteristics of the included studies are presented in Table 1.

**FIGURE 2.**
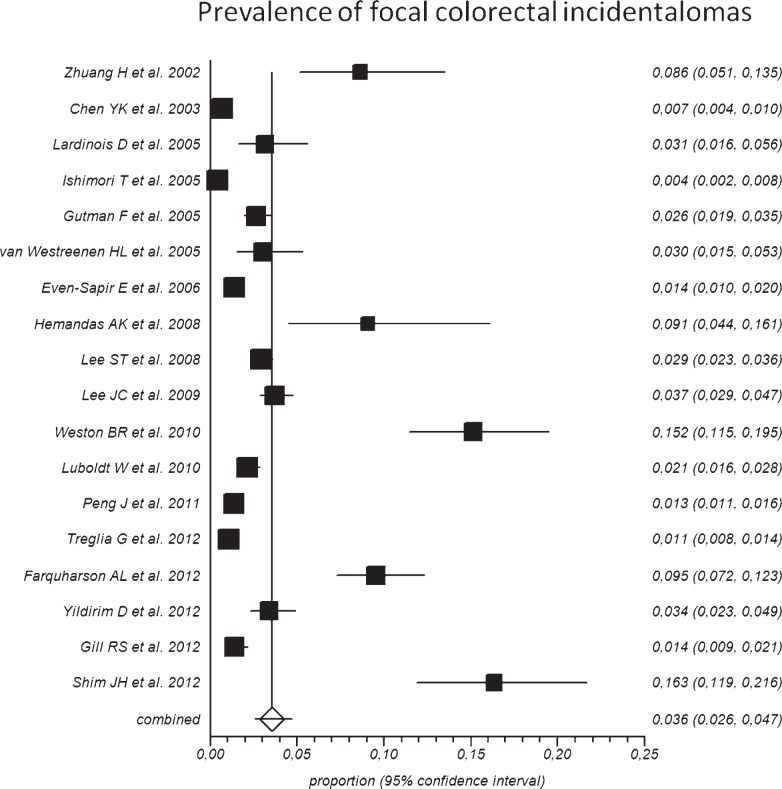
Plot of individual studies and pooled prevalence of patients with focal colorectal incidental uptake detected by ^18^F-FDG-PET or PET/CT, including 95% confidence intervals (95%CI). Prevalence of patients with focal colorectal incidental uptake ranged from 0.4% to 16.3%, with pooled estimate of 3.6% (95%CI: 2.6–4.7%). The included studies were statistically heterogeneous (I-square: > 75%).

**FIGURE 3.**
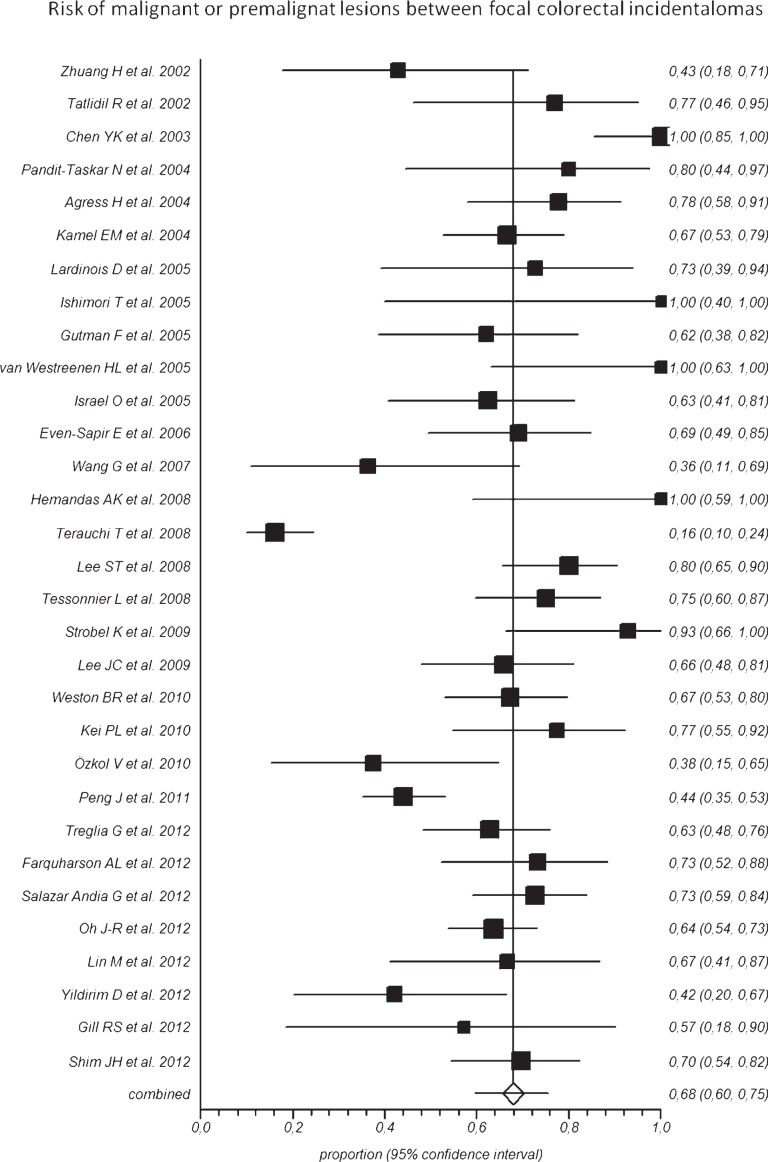
Plot of individual studies and pooled risk of malignant or premalignant lesions between focal colorectal incidental uptake detected by ^18^F-FDG-PET or PET/CT, including 95% confidence intervals (95%CI). The risk of malignant or premalignant lesions ranged from 16% to 100%, with pooled estimate of 68% (95%CI: 60–75%). The included studies were statistically heterogeneous (I-square: > 75%).

Overall, the pooled prevalence of FCIs detected by ^18^F-FDG-PET or PET/CT in the included studies was 3.6% (95% CI: 2.6–4.7%), ranging from 0.4% to 16.3% (Figure 2). Overall, 1,044 FCIs detected by ^18^F-FDG-PET or PET/CT underwent colonoscopy or histology verification. Pooled risk of malignant or premalignant lesions between FCIs was 68% (95% CI: 60–75%), ranging from 16% to 100% in the included studies (Figure 3). The included studies were statistically heterogeneous (I-square: > 75%) both for prevalence and risk of malignant or pre-malignant FCIs.

Concerning geographic distribution, the pooled risk of malignant or premalignant lesions in FCIs was lower in Asia-Oceania (62%; 95% CI: 43–79%) compared to America (70%; 95% CI: 61–79%) and Europe (70%; 95% CI: 65–74%).

A statistically significant difference in average SUV between malignant, premalignant and benign FCIs was reported in some articles; nevertheless, a significant overlap about SUV was found between these three groups (Table 1).

## Discussion

The increasing use of ^18^F-FDG-PET and PET/CT is associated with a concomitant increase in the number of patients with FCIs. The major difference between PET/CT and other imaging studies is that PET/CT provides both anatomic and metabolic information about incidental lesions found in the large bowel. The pattern of ^18^F-FDG uptake in the large bowel on PET imaging influences the likelihood of malignancy. Diffuse and segmental increased uptake detected at ^18^F-FDG-PET or PET/CT in the large bowel are usually associated with benign conditions 1,2: such cases were not covered by this meta-analysis. We focused our analysis on FCIs because they can be associated with malignant or premalignant conditions in a significant number of cases.^1^,^2^

Several single-center studies have reported the prevalence of FCIs and risk of malignant and premalignant lesions between FCIs detected by ^18^F-FDG-PET or PET/CT with discordant findings.^2^,^5^–^35^ In order to derive more robust estimates and obtain evidence-based data about this topic, we performed a meta-analysis pooling published data.

Pooled results of our meta-analysis indicate that FCIs are observed in about 3.6% of patients performing ^18^F-FDG-PET or PET/CT. Moreover, in our pooled analysis FCIs were associated with a high risk of malignant or premalignant lesions (68%), considering colonoscopy or histology confirmation as reference standard. Therefore, whenever a focal hot spot is detected within the large bowel, the ^18^F-FDG-PET or PET/CT report should suggest further investigation, such as colonoscopy, in order to exclude a malignant or premalignant lesions.^1^,^2^

In the calculation of pooled malignancy risk, we considered premalignant lesions together with malignant lesions because colonic adenomas can transform from adenoma to carcinoma and progress insidiously in asymptomatic patients.

Performing a sub-analysis for geographic areas we found that the risk of malignant or premalignant lesions between FCIs was higher in America and Europe compared to Asia and Oceania. A possible explanation of this finding is that the prevalence of colorectal cancer is superior in these geographic areas.^36^

A significant difference in average SUV between malignant, premalignant and benign FCIs was reported in some articles (Table 1). Nevertheless, a significant overlap about SUV was found between these three groups. Therefore, SUV alone should not be used to differentiate between malignant, premalignant and benign FCIs. Indeed, it is well known that SUV is influenced by several factors, related to the patient as well as to technical aspects and procedures. Any calculation of a pooled SUV obtained by different studies - acquired with different tomographs, scan protocols, ^18^F-FDG injected activity, and patient characteristics - is in our opinion inappropriate, and therefore we decided not to meta-analyze data about SUV.

The present study has some limitations, related to the included articles, such as the selection bias in the calculation of malignancy risk and the heterogeneity between studies. Indeed, only a percentage of FCIs detected by ^18^F-FDG-PET or PET/CT underwent colonoscopy or histopathology confirmation in the included studies and this may represent a selection bias in the calculation of the risk of malignant or premalignant lesions. Furthermore, the included studies were statistically heterogeneous in their estimates of prevalence of FCIs and risk of malignant or premalignant lesions. This heterogeneity is likely to stem from diversity in methodological aspects between different studies. The baseline differences between the patients performing PET or PET/CT in the included studies may have contributed to the observed heterogeneity too. However, such variability was accounted for in a random-effects model.

Lastly, we did not perform a sub-analysis taking into account the device used (PET vs. PET/CT) or the site of FCIs (rectum and different colonic segments) because sufficient data in this regard could not be retrieved from the included studies.

## Conclusions

FCIs are observed in a not negligible number of patients who undergo ^18^F-FDG-PET or PET/CT studies with a high risk to be malignant or premalignant lesions. SUV is not reliable as a tool to differentiate between malignant, premalignant and benign FCIs. Further investigation, such as colonoscopy, is warranted whenever FCIs are detected by ^18^F-FDG-PET or PET/CT in order to exclude malignant or premalignant lesions.

## Figures and Tables

**TABLE 1.** Characteristics of the included studies about focal colorectal incidental uptake detected by ^18^F-FDG PET or PET/CT

**Authors**	**Year**	**Country**	**Device used**	**No. of patients evaluated**	**No. of patients with FCIs**	**No. of FCIs**	**No. of FCIs verified by colonoscopy or histology**	**Final diagnosis of FCIs**				**Average SUV in FCIs**		
								**Malignant**	**Pre-malignant**	**Benign**	**No lesions identified**	**Malignant FCIS**	**Pre-malignant FCIs**	**Benign FCIs**
Zhuang H et al.	2002	USA/Brazil	PET	197	17	17	14	5	1	8		n.a.	n.a.	n.a.
Tatlidil R et al.	2002	USA	PET	3000	n.a.	n.a.	13	6	4	3	0	n.a.	n.a.	n.a.
Chen YK et al.	2003	Taiwan	PET	3210	22	23	23	6	17	0	0	5.74 ± 2.26^*^	3.56 ± 0.68^*^	n.a.
Pandit-Taskar N et al.	2004	USA	PET	1000	n.a	n.a.	10	1	7	0	2	13.6	7.0 ± 3.0	n.a.
Agress H et al.	2004	USA	PET	1750	n.a.	n.a.	27	3	18	3	3	n.a	n.a	n.a.
Kamel EM et al.	2004	Switzerland	PET/CT	3281	n.a.	n.a.	54	9	27	9	9	n.a	n.a	n.a.
Lardinois D et al.	2005	Switzerland/Russia	PET/CT	350	11	11	11	0	8	3	0	n.a.	n.a	n.a.
Ishimori T et al.	2005	USA	PET/CT	1912	8	8	4	4	0	0	0	n.a.	n.a.	n.a.
Gutman F et al.	2005	France	PET/CT	1716	45	n.a.	21	3	10	1	7	15 ± 11.6	12.0±3.7	25
van Westreenen HL et al.	2005	The Netherlands	PET	366	11	11	8	2	6	0	0	n.a.	n.a.	n.a.
Israel O et al.	2005	Israel	PET/CT	4390	n.a.	n.a.	24	6	9	3	6	n.a.	14.0 ± 10.5	n.a.
Even-Sapir E et al.	2006	Israel	PET/CT	2360	33	39	29	13	7	5	4	n.a	n.a	n.a.
Wang G et al.	2007	China/Australia	PET/CT	1727	n.a.	n.a.	11	1	3	4	3	n.a.	n.a.	n.a.
Hemandas AK et al.	2008	UK	PET/CT	110	10	10	7	0	7	0	0	n.a.	n.a.	n.a.
Terauchi T et al.	2008	Japan	PET	2911	n.a.	111	111	7	11	9	84	8.31	n.a.	n.a.
Lee ST et al.	2008	Australia	PET/CT	2916	85	95	45	12	24	2	7	n.a.	n.a.	n.a.
Tessonnier L et al.	2008	France	PET/CT	4033	n.a.	n.a.	44	8	25	4	7	12.3 ± 5	9.8 ± 6.1	8.2±2.1
Strobel K et al.	2009	Switzerland	PET/CT	598	n.a.	14	14	5	8	0	1	n.a.	n.a.	n.a.
Lee JC et al.	2009	Australia	PET/CT	1665	62	70	35	11	12	5	7	n.a.	n.a.	n.a.
Weston BR et al.	2010	USA	PET/CT	330	50	52	52	10	25	2	15	17.2^*^	14.2 ± 7.2^*^	n.a.
Kei PL et al.	2010	USA/Singapore/Hong Kong	PET/CT	2250	n.a.	n.a.	22	4	13	1	4	n.a.	20.7 ± 11.3^*^	12.0^*^
Özkol V et al.	2010	Turkey	PET/CT	2370	n.a	n.a.	16	3	3	7	3	n.a.	n.a.	n.a.
Luboldt W et al.	2010	Germany	PET/CT	2338	50	n.a.	n.a.	n.a.	n.a.	n.a.	n.a.	n.a.	n.a.	n.a.
Peng J et al.	2011	China	PET/CT	10978	148	n.a.	125	32	23	5	65	9.7^*^	8.2^*^	6.1^*^
Treglia G et al.	2012	Italy	PET/CT	6000	64	n.a.	51	13	19	8	11	9.6 ± 4.7	8.5 ± 5.2	6.5 ± 3.6
Farquharson AL et al.	2012	UK	PET/CT	555	53	n.a.	26	2	17	3	4	n.a.	n.a.	n.a.
Salazar Andia G et al.	2012	Spain	PET/CT	2220	n.a.	n.a.	55	13	27	10	5	n.a.	n.a.	n.a.
Oh J-R et al.	2012	Republic of Korea	PET/CT	21317	n.a.	296	102	32	43	13	14	13.6 ± 4.9^*^	8.4 ± 4.5^*^	6.8^*^
Lin M et al.	2012	Australia	PET/CT	649	n.a.	n.a.	18	3	9	4	2	6.0	10.4	5.8
Yildirim D et al.	2012	Turkey	PET/CT	823	28	28	19	6	2	1	10	n.a	n.a	n.a.
Gill RS et al.	2012	Canada	PET or PET/CT	1500	21	21	7	2	2	1	2	7.4	n.a.	4.1
Shim JH et al.	2012	South Korea	PET/CT	239	39	46	46	8	24	14		8.9	5.5	n.a.

FCIs = focal colorectal incidental uptake; pts = patients; n.a. = not available;

* significant statistical difference
